# How German general practitioners justify their provision of complementary and alternative medicine – a qualitative study

**DOI:** 10.1186/s12906-020-02907-6

**Published:** 2020-04-15

**Authors:** Agnes Ostermaier, Niklas Barth, Klaus Linde

**Affiliations:** 1 Technical University of Munich, TUM School of Medicine, Institute of General Practice and Health Service Research, Orleansstrasse 47, D-81667 Munich, Germany; 2 Ludwig-Maximilans-Universität Munich, Institute of Sociology, Konradstr. 6, D-80801 Munich, Germany

**Keywords:** Primary care, General practice, Complementary and alternative medicine, Legitimization

## Abstract

**Background:**

Many German general practitioners (GPs) use complementary and alternative medicine (CAM) in their daily work although most CAM procedures are controversial from an academic point of view.

**Objective:**

We aimed to investigate how GPs justify their use of CAM.

**Methods:**

We performed semi-structured, individual face-to-face interviews with 20 purposively sampled, experienced GPs providing primary care within the framework of the German statutory health insurance system. A grounded theory approach was used for data analysis.

**Results:**

All GPs participating in this study used at least some CAM in their clinical practice. Participants did not have any major conflicts when justifying their use of CAM therapies. Important arguments justifying CAM provision were: using it as a supplementary tool to conventional medicine; the feeling that evidence and science leave many problems in primary care unanswered; a strong focus on helping the individual patient, justifying the use of procedures not based on science for therapeutic and communicative purposes; a strong belief in one’s own clinical experience; and appreciation of placebo effects. In general, participants preferred CAM therapies which seemed at least somewhat plausible to them and which they could provide in an authentic manner.

**Conclusions:**

Our results suggest that many German GPs integrate CAM treatments in their routine primary care work without perceiving any major internal conflicts with professional ideals.

## Introduction

Most complementary and alternative medicine (CAM) treatment modalities are scientifically controversial and seen with great skepticism by academic medicine [1, 2]. Yet, quantitative surveys show that in a number of countries a substantial proportion of conventionally trained physicians apply, prescribe or recommend CAM in clinical practice, either alongside or in place of conventional medicine, with general practitioners (GPs) being particularly open to such therapies [3–9]. These surveys suggest that many GPs believe that CAM can be associated with positive effects and some firmly believe in the efficacy of specific therapies [3, 5, 7, 9]. A good safety profile, more “holistic” approach, lack of response to conventional treatment, and limitations in the scientific worldview are other frequently reported reasons for CAM provision. In Germany, the use of CAM among GPs seems to be particularly prevalent [6, 9]. Eighty-five percent of GPs participating in a national survey [5] reported using at least one CAM treatment modality once weekly or more often in clinical practice, with herbal remedies (77%), vitamins and supplements (41%) and homeopathic remedies (32%) being most frequent.

A variety of cultural and historic factors might contribute to the widespread use of CAM treatments among German physicians in general and among GPs in particular. The popularity and use of CAM modalities among the German population is high [10] and there is a long-standing tradition of interest into non-conventional and “natural” treatments both in the general population and among physicians [11]. The German Medical Association accredits so-called additional qualifications (*German* “Zusatzbezeichnungen”; see Additional File 1 for further information) for several CAM therapies such as acupuncture, homeopathy, natural healing procedures (including herbal medicines, spa therapy etc.) and manual medicine/chirotherapy (including a variety of manipulative, chiropractic and osteopathic techniques). Herbal, homeopathic and anthroposophic medicines are regulated as drugs and sold in pharmacies.

Health care for 90% of the German population is covered by social health insurance (88%) or by related funds (2%), 10% are privately insured. Compared to other countries, the German health system has a relatively strong market-orientation, the workload of GPs is high and average contact times per patient visit are very short within the social health insurance system [12]. Most CAM is not covered by social health insurance, but there is a great willingness in the population to pay privately for such treatments [13]. Thus, providing CAM can also be financially attractive for GPs. Furthermore, the freedom of the physician to choose a therapy has traditionally been an important professional principle for many German physicians [14]. Yet, CAM has little role in medical education and skeptical academics criticize the broad CAM provision as an unacceptable deviation from evidence- and science-based medicine [2].

We have performed a qualitative study among experienced German GPs to better understand why they use CAM treatments as well as other professionally questionable interventions (for example, antibiotics in patients with the common cold). In an earlier article [15] we described our findings suggesting that GPs generally use such strategies to manage “therapeutically indeterminate situations”. Such situations are characterized by two sets of conditions: “firstly, there is a desire for medical treatment, either by the patient or the physician, or both. Secondly, either such a treatment is not (unambiguously) necessary from a medical perspective, a professionally accepted treatment is not available, or an existing effective treatment is not acceptable to the patient” (p2 [15]). Therapeutically indeterminate situations occur frequently in patients with minor illnesses, medically unexplained symptoms, or long-lasting complaints associated with chronic diseases. Beside empathic consultations, the use of CAM was an important tool for managing these situations. In this article, we report how participants justified their use of CAM as GPs and whether they perceived such use a problematic deviation from professional ideals.

## Methods

As the methods of our study have been described in detail elsewhere [15], we only give a relatively brief description below. After providing informed consent, purposefully sampled experienced GPs providing first-line primary care in Bavaria, Germany, participated in problem-oriented interviews (performed between July 2015 and December 2016). We aimed to recruit similar numbers of GPs belonging to the following groups: those who exclude CAM treatments as far as posible, those who use CAM treatments occasionally but without being convinced of their specific effects, and convinced CAM providers. This information was collected through pre-study contact, websites and information from colleagues. Furthermore, we took account of gender and practice location. We did not include physicians trained as GPs but not participating in the social health insurance primary care system or exclusively offering specialized CAM care.

All interviews were conducted face-to-face with a single GP (19 by AO and one by NB and KL). Median duration was 52 min (range 34 to 72 min). While an interview guide listing issues to be addressed was available (see Additional File 1) we aimed to get the interviewees to develop the themes themselves as much as possible. Topics to be addressed included typical features of their practice and how they had changed over the years; attitudes to and use of CAM treatments, non-specific treatments and placebos, and if such treatments were not used, how practice was managed without them; scientific orientation, justification of the strategies used and inner conflicts; and their views on their role as a physician and medical doctor. Interviews were audio-taped, transcribed verbatim, and pseudonymized.

A grounded theory approach was used for data analysis ([16, 17], see Additional File 1 for further details). Analysis started early in the data collection phase. The coding process used elements of open, axial and selective coding and was supported by writing memos and developing diagrams. Coding, interim findings and our interpretations reflecting our prior and adapted assumptions were regularly discussed by the research team.

## Results

### Charateristics of participants

A total of 20 GPs participated in the study. Ten participants were between 41 and 50 years old, five between 51 and 60 and another five between 61 and 70 years. Eight GPs were female, twelve male. Four practices were based in cities or suburbs, ten in towns and six in villages. Fourteen participants had been specialized as GPs for more than 10 years, and 18 were either the only practice owner or a joint partner. Nine had an additional qualification in at least one CAM therapy (6 acupuncture, 5 naturopathy, 5 homeopathy, 2 chirotherapy) and eight an additional qualification in emergency medicine.

While several participants considered themselves skeptical towards CAM, all prescribed at least herbal remedies in their clinical practice. Based on their general tendencies in the interview, we considered only one participant a true skeptic (i.e., generally believing that CAM treatments do not have any effects over placebo and trying to avoid such treatments as far as possible) and four as clearly convinced CAM providers (i.e., firmly believing that the CAM treatments used have specific effects). The remaining participants seemed to be highly pragmatic in their views on CAM, ranging from rather skeptical to very open.

### The basis is conventional medicine - CAM is a supplementary tool

One important indirect argument made by participants legitimizing the use of CAM was that they use these treatments largely as a practical supplement when conventional medicine does not provide satisfactory solutions. Most participants (including convinced CAM providers) explicitly emphasized that conventional medicine is the basis of their clinical practice.

*Complementary medicine is a very good supplement, but only up to a certain point. The core should be conventional medicine, around which other things from here and there can be used as complements. It’s a good compromise, that's how I see it. The basis is conventional medicine, to which other things can be added. (17)*

Two GPs using CAM intensively and in a convinced manner in their clinical practice even stated that, for them, being competent in conventional medicine is a precondition to apply these modalities.

*My thinking was I have to be able to practice conventional medicine properly in order to justify my use of other things. (11)*

### The limits of evidence and science in general practice

At the same time, participants reported that conventional medicine with its focus on evidence and science leaves many problems in primary care unanswered (see our article on therapeutically indeterminate situations [2] for details). In the following quote, a participant who originally specialized in internal medicine explained why CAM modalities are “dispensable” in specialized care but can be helpful in general practice, despite classifying himself as rather skeptical. The last phrase of the quote is also an example of how participants started to question their own positions during the interviews which addressed issues rarely discussed openly.

*I’m sure that if I had a specialist practice as an internist, where you really get referrals with clear questions, complementary medicine is dispensable, or a very small niche, as in irritable bowel syndrome or whatever, or with palpitations and things like that at the cardiologist, where you might try something, but otherwise rarely. But with us [in general practice], I think, because it's such a diffuse, unsorted patient population, I find that you do actually need it. Well, right now I'm sticking my neck out in favor of complementary medicine although I told you at first that I don’t use it at all, so I’m not sure I belong in this interview any more. (13)*

Participants using CAM more frequently argue that the quality of evidence in general practice is often weak.

*It's just that if you go a little deeper into evidence-based medicine, you'll see that even in conventional medicine, the evidence is sometimes astonishingly thin. (03)*

These participants also tend to address concerns regarding unclear or implausible mechanisms of action for CAM treatments by pointing to gaps in mechanistic knowledge in conventional medicine and the fact that current knowledge is often incomplete and sometimes falsified by new research.

*It is often the case with conventional drugs, too, that one doesn’t really understand the mechanism of action, or only vaguely what’s happening. And every few years there's something new, new knowledge. (06)*

In general, science with its empirical principles based on populations rather than individuals was not considered to be the only guide for daily work in general practice.

*The problem is that people are always different, that ... all science ultimately forgets the individual, and so the actual individual case. (15)*

### Helping individual patients without doing harm

Participants felt primarily committed to helping the presenting patient.

*… it is always about the individual patient and their problem … (16)*

To have practical solutions in as many situations as possible and in order to be able to take account of the preferences of patients, participants aimed to have broad toolkits.

*The benefit is that I simply have a broader repertoire … Where conventional medicine has its gaps or where the patient has other ideas, I still have therapeutic alternatives. (03)*

They judged the different therapies by their contribution to the potential solution of the patient’s problem. The means to reach the therapeutic goals were considered secondary. In some situations CAM could become an alternative instead of a supplement for more convinced CAM providers.

*In my opinion, one has a therapeutic goal in each case. And I stick to it. And I do my best to achieve it. And it doesn’t matter how I achieve it. So, if the man can walk again, because the pain in the hip has diminished, then the therapeutic goal has been reached. It doesn’t matter whether the hip has been replaced or if I’ve used acupuncture it or recommended something else. (04)*

*Complementary medicine is sometimes even the better tool for me, not so as to avoid patients going somewhere else, but because in my eyes I can better help them with this kind of medicine. For that reason, I would even prefer complementary medicine in certain cases. (14)*

Participants often prescribed CAM treatments to avoid conventional treatments with a higher risk of side effects, but also stressed that the use of CAM itself must not harm the patient.

*For a cold, I prefer to give the patient only a [homeopathic] remedy, which has no side effects, instead of saying, now take ibuprofen plus [brand name for acetylcysteine] plus blah blah, which … according to the evidence provides little benefit and maybe also has side effects. (07)*

*The main thing is not to harm the patient. Also not by losing time or whatever, you have to bear that in mind. (03)*

### Facilitating communication and the therapeutic relationship

The motives for using CAM to facilitate empathic communication and to develop a better relationship with patients arise in various ways in the interviews, particularly among GPs seeing many patients open to CAM therapies. For example, when talking about his “broad toolkit” the participant cited in the previous section also said that his CAM skills and experience allow him to take a more complete case history.

*I am more open-minded when I speak with patients, and can take a better case history. I can better understand what other treatments the patient has already undergone, or is undergoing. (03)*

The openness of the GP to CAM could increase the trust of patients skeptical toward conventional medicine when a conventional treatments are really needed:

*Or when I prescribe an antibiotic they say: "If that’s what you say, then I know I really need it. If I were at the ENT [ear, nose, throat] doctor’s, I wouldn’t take it, but if you say I need it, then I really do need it, because I know that you’d prefer not to give it to me." (07)*

CAM was also considered to be a means of accompanying patients with severe diseases or in difficult social situations.

*Of course, a big area is the people with cancer who actually come to see me because of the mistletoe, because they’ve heard I prescribe it. And then you have to see why they actually come. … And then you have to listen carefully and look, talk to the patients … if they really want the mistletoe or if they’re just running away from their disease. And it often is the case that they’re running away. And then I try to accompany them on their way and the goal is to provide them with effective therapy. And that often means having surgery and, if necessary, doing chemotherapy and, of course, radiotherapy. But for me, the most important thing is to build trust with these patients, and to strengthen their self-confidence and courage, so that they cope with the disease, that’s the most important thing. I cannot say, “I'll give you the mistletoe and you’ll be all better”, that's not it, it's more about accompanying them on their way. (16)*

### Confidence in own practical experience

In general, all participants strongly trusted their personal practical experience. But while a few more skeptical GPs emphasized that personal experience is not sufficient for assessing whether a CAM treatment has specific effects, positive experiences were sufficient for the majority of participants to become open to CAM provision. These GPs judged the usefulness of CAM (as well as of conventional) treatments based primarily on what they saw in their patients. Rising doubts about the specificity of effects or the plausibility of potential mechanisms were overturned by the repeated experience of positive outcomes.

*The decisive factor is the effect. So what matters is the outcome and not necessarily knowing how it works ... we see that it works, and then I can administer it as long as it doesn’t harm the patient. (15)*

*I do have my doubts and wonder what it is that works … but then I often see these amazing reactions, and then I say, there must be something in it. (14)*

### Appreciation of placebo effects

Whether placebo effects had a role when using CAM was not a major issue for participants. On the contrary, placebo effects were appreciated as an important and positive component of any treatment.

*Of course, the placebo effect is very important. It should not be underestimated, but many conventional doctors do just that: "That's just a placebo". Placebo is really quite remarkable. (06)*

Yet, when reporting on their personal use of CAM treatments participants usually implicitly or explicitly stated that they did not think that these treatments work *exclusively* by placebo effects (note the word “partly” in the following quote).

*If the patient is happy and well, then that’s fine by me … whether it’s partly a placebo effect or not is all the same to me. (07)*

### Personal beliefs and the need to be authentic

If, instead, participants considered a CAM treatment to work *exclusively* through placebo effects, this usually had the consequence that they did not use it. The GP quoted below considered homeopathy a “great placebo”, but when trying to use it he felt as though he lost his authenticity and was lying to the patient. So he preferred using herbal remedies which were more plausible to him even if convincing clinical evidence from trials was missing.

*[Homeopathy] is a great placebo. Really, there is really hardly anything in which one orients so much to the patient and ... where everything has meaning, and then one chooses the appropriate remedy. It's really astounding, how much there is to it. ... But I can’t hand on heart do something I don’t really believe in, so, yes, I've actually thought, ok, take it as a placebo … but then I really felt I was lying, playing a part, and that's not authentic. Exactly. That's why it didn’t work for me ... And with the other remedies, such as herbal remedies, there is at least an active ingredient, even if there’s no evidence from clinical trials. (13)*

Based on their personal beliefs, preferences and experiences, participants made quite individual choices of CAM therapies for clinical use.

*I have experience with acupuncture and Ayurveda. Therefore, I know they work. … As for homeopathy, I have no use for it. (19)*

Plausibility and evidence of effectiveness – as perceived by the individual physician – influenced whether a treatment was considered acceptable or not. Herbal medicines seemed acceptable to almost all participants, while homeopathy was often discussed critically.

*And then there’s homeopathy, which I haven’t talked about at all, because I never use it ... for me it’s completely irrational to think that this has any effect (laughs). And there are no significant proven effects. Although the homoeopaths always say there are. It wouldn’t occur to me. (05)*

### Individual strategies avoiding internal conflicts

In summary, over the years the experienced GPs in our study had developed individual strategies integrating personally selected CAM therapies to a greater or lesser extent into their daily clinical practice. These individual strategies were constructed in a manner which minimized internal conflict and supported the desire to provide good primary care.

*… whether it’s morally justifiable, I have to say that personally it’s simply not an issue, because – and this applies to all medicine for me - I try to help the patient and avoid harming them, with all the means at my disposal. … The goal is to find the best possible solution for the patient. (03)*

## Discussion

### Summary of findings

All the experienced German GPs participating in this study used at least some CAM in their clinical practice. Using CAM did not cause any major conflicts with professional ideals. Important arguments justifying CAM provision were: using it as a supplementary tool to conventional medicine; the feeling that evidence and science leave many problems in primary care unanswered; a strong focus on helping the individual patient, also justifying the use of procedures not based on science for therapeutic and communicative purposes; a strong belief in their own practical experience; and an appreciation of placebo effects. Participants preferred CAM therapies which seemed at least somewhat plausible to them and which they could provide in an authentic manner.

### Comparison with existing literature

There is only a small number of qualitative studies systematically investigating why conventionally trained GPs (two studies also included other physicians) engage with CAM. The specific issues addressed in these studies varied considerably. Four of these studies (two from Germany, one each from Australia and the Netherlands) exclusively included frequent CAM providers [18–22], and it is unclear to what extent the participants provided true primary care (i.e., first contact and principal point of continuing care for patients within a healthcare system) or more some sort of CAM-focused care attracting a specific selection of patients. The two remaining studies from New Zealand and the United Kingdom recruited GPs providing primary care regardless of CAM provision, but were performed in countries in which *active* CAM provision is rare among this group of physicians [23, 24]. Our study adds another piece to the puzzle as it recruited primary care providers in a country with frequent CAM provision among GPs.

Several basic findings are similar across the studies. For example, consistently reported reasons for engaging with CAM were the importance of the physician’s belief in efficacy, the belief that there are fewer side effects, and the feeling that science leaves many practical problems unanswered. In contrast to the qualitative studies conducted in other countries, the integration of CAM into routine primary care was more deeply embedded into the clinical practice of the participating GPs in our study. Herbal medicines seem to be almost conventional medicine to many German physicians and were recommended by all our participants. Furthermore, the majority of participants integrated combinations of several CAM therapies into their daily practice without perceiving any major professional conflicts. These findings are in contrast to the studies in GPs providing primary care in New Zealand or the United Kingdom. The study from New Zealand [23] describes the participants’ “discomfort” regarding the “gulf between the two paradigms”. The study from the United Kingdom [24] focuses on beliefs about utility but the findings suggest that the active use of CAM among participants was very limited. While participants in both studies saw some role for CAM in clinical practice, concerns about evidence seemed to be more pronounced than in our study and the intrinsic motivation for actively using CAM was much lower. Further systematic investigation of historical, cultural and professional factors is required to investigate and explain the observed differences..

Compared to the studies exclusively recruiting physicians with intense CAM provision [18–22], the majority of participants in our study seemed clearly more biomedically oriented. According to a categorization proposed by Frank and Stollberg [20] most of our GPs seem to practice a model of “biomedically dominated coexistence” of “orthodox” and “heterodox” medicine, “in which isolated heterodox techniques are integrated into the therapeutic arsenal, while the dominance of biomedical concepts remains unchallenged” (page 357). A few of our more CAM-convinced participants might be on the border of a “coexistence with heterodox dominance” where CAM becomes the preferred approach. Frank and Stollberg also described a group (which was not observed in our study) with a strong CAM focus who integrate several traditions of healing (for example, Ayurveda, traditional Chinese Medicine and homeopathy) in a “great melting pot” (page 358). None of our participants seemed to practice such a model.

Frank and Stollberg developed their categorization in a study of medical acupuncturists, only some of whom were GPs. Therefore, their categorization might not be fully adequate for differentiating GPs providing CAM within a social health insurance system. A variety of qualitative studies show that many GPs feel that biomedicine does not provide satisfying answers for a significant proportion of the patients they see (e.g. [15, 25–28]). Furthermore, managing uncertainty is a crucial skill for primary care [29, 30]. Yet, biomedicine does not seem to have been actively challenged by any of the participants in these studies. Differentiating subtypes of the model of “biomedically dominated coexistence” could be a worthwhile objective for future studies.

Another interesting empirical finding of our study is the important link between being able to act in an authentic manner, the exploitation of placebo effects and the personal belief in specific effects. Our findings fit with those in the scarce qualitative literature on prescribing placebos which suggests that exploiting the placebo effects of a treatment seems to be fully legitimate only if the provider believes that it also has specific intrinsic effects [31, 32]. Parsons identified functional specificity as a core value of the medical profession [32, 33]. In her seminal study among Welsh GPs from 1976 [25], Comaroff described how physicians internalize the professional ideal to provide treatment only if needed and only if it has specific effects (over placebo). If a physician is completely certain that a CAM treatment is a placebo, it becomes illegitimate for this physician.

From a sociological perspective, the use of CAM strategies by GPs also draws attention to a central aspect of the doctor-patient interaction. Talcott Parsons has attested that medicine has an “optimistic bias” [25]. The suffering of a patient almost urges the attending physician to a therapeutic action. This implies that any act that could help to alleviate the patient’s suffering is considered more legitimate than doing nothing. Our data show that CAM procedures seem to be one functional solution [15] to the problem of the doctor being under pressure to act professionally. On the one hand, this pressure to act is institutionalized by the fact that patients approach doctors with an expectation of therapeutic action. On the other hand, the treatment of patients is shaped by the professional ethic that implies that patients should at least not be harmed. CAM strategies seem to offer a means of navigating between these imperatives. Under conditions of uncertainty in daily primary care, they can represent a resource to assist the physician’s basic ability to act. They enable doctors to better connect with patients while also avoiding harm.

### Limitations

Our small study cannot make any claims for generalizability, but our findings fit well with those of larger quantitative surveys among German GPs [5, 6, 9]. Our findings are strongly influenced by the specific cultural and health service conditions in Germany. Furthermore, our study has been performed in the southern part of Bavaria, a region with income above the German average. Although valid data is not available it seems likely that the use of CAM among the general population in this region is also above average. Obviously, the reports of our participants are active retrospective re-constructions of what they consider their actual practice. Social desirability and reducing contradictions might have influenced the accounts of GPs. As our findings show, participants have individually differing views and beliefs on different CAM methods. Focusing on a single CAM modality would have sharpened findings for this specific treatment. However, we aimed to address the subject in a broader manner to better understand how German GPs use CAM treatments in their primary care work in a more general manner.

## Conclusions

Our results suggest that many German GPs integrate CAM treatments in their routine primary care work without perceiving any major internal conflicts with professional ideals. As the level of integration of CAM into general practice seems to differ strongly between countries, international cooperation to explore influencing factors would be desirable in future projects.

## Supplementary information

**Additional file 1.**

## Data Availability

Data collected and analyzed during the current study are not publicly available due to the confidential nature of participant transcript data. AO’s thesis with a comprehensive collection of quotations (in German) will be available from the authors after approval by the Medical Faculty of the Technical University of Munich.

## Abbreviations

CAM: Complementary and alternative medicine
ENT: Ear, nose, throat
GP: General practitioner
