# Supplementary material for: How German general practitioners justify their provision of complementary and alternative medicine – a qualitative study
Source: BMC Complement Med Ther. 2020 Apr 15;20:111. doi: 10.1186/s12906-020-02907-6 (PMC7158128; doi:10.1186/s12906-020-02907-6)
Supplement: Supplementary file 1 — Additional file 1. [file 12906_2020_2907_MOESM1_ESM.docx]

Ostermaier et al. How experienced German general practitioners justify their use of complementary and alternative medicine – a qualitative study

**ADDITIONAL FILE 1**

**I Information regarding the additional qualifications accredited by the regional
 chapters of the GMA (German Medical Association)**

**II Topic guide**

**III Additional details regarding analysis and translation of quotes**

**I - Information regarding the additional qualifications accredited by the regional chapters of the GMA (German Medical Association)**

**Acupuncture** Additional qualification currently certified after 200 hours of training (120 hours training, 20 hours case presentation seminars, and 60 hours acupuncture practice supervised by an accredited practitioner.^1^ Number of certified physicians Dec 2018: 13,198 (out of a total 392,400 active physicians)/ 11,498 (out of a total of 157,300 physicians providing outpatient care) ^2^

**Homeopathy** Additional qualification currently certified after 160 hours training, plus 100 hours case presentation seminars or six months homeopathy practice supervised by an accredited practitioner.^2^ Number of certified German physicians Dec 2018: 5,482 / 4,665 ^3^

**Manual Medicine/Chirotherapy** (covering a variety of manipulative, chiropractic and osteopathic techniques) Additional qualification currently certified after 320 hours of training.^2^ Number of certified German physicians Dec 2018: 17,989 / 13,278 ^3^

**Natural Healing Procedures** (‘Naturheilverfahren’ includes a number of traditional therapies such as phytotherapy (use of herbal medicines), spa and climate therapy, exercise, breathing and relaxation techniques; massage and reflexology; nutritional and fasting techniques; lifestyle approaches; physician therapies;) Additional qualification currently certified after 160 hours of training, plus 100 hours case presentation seminars or three months work with a certified homeopathic physician or 100 hours supervised case seminars.^2^ Number of certified German physicians Dec 2018: 16,111 / 10,856 ^3^

1 <https://www.blaek.de/weiterbildung/qualifikationen-nach-der-weiterbildungsordnung>

2 <http://www.gbe-bund.de/>

**II - Topic Guide**

**Theme 1: The practice**

Guiding questions: Can you tell us a bit about your practice? Do you have any specialisations regarding patients and therapies? What has changed over the years?

**From here on, address themes in any sequence, or only if they do not come up spontaneously**

**Theme 2: Complementary therapies**

Guiding questions: Do you use complementary therapies in your practice (e.g., homeopathy, herbal medicines, acupuncture etc.)?

Additional questions when the answer is ‘yes’: To what extent? How relevant are these therapies for you? Do you think they are effective? Are they specifically effective or more a placebo therapy? Do think this is scientifically or medically justifiable?

Additional questions when the answer is ‘no’: How do you manage your practice without these therapies, which are frequently used by your colleagues? Why do they need these therapies while you don’t?

**Theme 3: Placebos and non-specific treatments**

Guiding questions: Have you ever used a placebo (e.g. a placebo pill; or a saline injection)? Have you ever used a treatment which you did not consider effective (e.g., a homeopathic remedy if you consider homeopathy a placebo therapy) or one which you felt was not indicated (e.g., an antibiotic for a common cold)?

Additional questions when the answer is ‘yes’: Can you give examples? Why did you do it?

Additional question when the answer is ‘no’: How do you manage your practice without?

**Theme 4: Science**

Guiding questions: Which role do science and scientific evidence have in your practical work?

Additional questions, if needed, on the role of experience, plausibility, trials/meta-analyses, and whether or not you should refrain from a treatment more often?**III - Additional details regarding analysis and translation of quotes**

**Additional details regarding analysis**

All interviews were read by AO (female; at the time of the study a medical student with previous experience as a CAM practitioner) and KL (male, MD; a clinical epidemiologist with 25 years’ experience in quantitative research on CAM and placebo). A selection of interviews were also read by NB (male, PhD; a sociologist specialised in systems theory).

Our analysis was inspired by grounded theory (ref. #16 and #17 in the main text). Our primary perspective was functionalistic with the key question being: what problems are solved by the use of CAM, non-specific treatments and placebos. The evaluation framework of the interview data followed methodological principles of system-theoretical hermeneutics. Through the functionalist heuristic, narrative patterns were read as communicative solutions to self-imposed problematisations [1]. While our previous article (ref. #15 in the main text) had a stronger theory-building, synthetic approach, the article on hand is more descriptive.

Analysis started early in the data collection phase. In a first step, open coding was performed by AO. Early coding steps were supported by using MAXQDA 12 software. Units of coding could be single sentences but more often were several sentences to maintain (and code) structural references. The codes were continuously sorted and compared until categories with similar information emerged. If necessary categories were revised based on new data. In a second phase of axial coding, categories were consolidated and we tried to identify how categories related to each other, also with respect to the assumptions described above. Selective coding was not formally separated from axial coding, but in the third phase we aimed to construct a story line around the perception of indeterminateness and the functional strategies for dealing with it and justification of CAM use. Throughout the analysis, we followed a constant comparative approach. Transparency and trustworthiness in open coding was observed by the senior author who independently reviewed the coding framework, and subsequently through discussion with the sociologist. Written memos, reflective notes and mind maps supported the analysis. Findings were reported twice to groups of GPs to obtain feedback on the practical relevance of the categories developed.

References

1. Nassehi A, Saake I. Kontingenz: Methodisch verhindert oder beobachtet? Ein Beitrag zur Methodologie der qualitativen Sozialforschung. [Contingency: methodically eliminated or observed? A contribution to the methodology of qualitative research] Ztsch Soziol. 2002;31:66-86.

**Translation of original quotes into English**

Original quotes (in German language) selected for the manuscript were translated in a three-step approach into English. In the first step the senior author (KL) made a “raw” translation. Together with the German original quotes and a manuscript draft these were sent to a native (English) professional language editor for a first check. The corrected translations were then sent back to the senior author who, in case of any uncertainties, back-translated the quotes into German. This process was supported by electronic aids (translate.google.com and <https://www.linguee.de>). The language editor and the senior author then met and compiled the final translation. They tried to remain as close to the original German wording as possible, but sometimes the wording in the translations had to change considerably in order to convey the meaning of the participants’ colloquial statements.
